# Detection of HER-2/neu-positive circulating epithelial cells in prostate cancer patients

**DOI:** 10.1038/sj.bjc.6601532

**Published:** 2004-01-20

**Authors:** N Ady, L Morat, K Fizazi, J-C Soria, M-C Mathieu, D Prapotnich, L Sabatier, L Chauveinc

**Affiliations:** 1 CEA, Laboratoire de Radiobiologie et Oncologie, DRR/DSV, 92265 Fontenay-aux-Roses, France; 2 Département de Médecine, Institut Gustave Roussy, 94805 Villejuif, France; 3 Département d'Anatomopathologie, Institut Gustave Roussy, 94805 Villejuif, France; 4 Service d'Urologie, Institut Mutualiste Montsouris, 75014 Paris, France; 5 Département de Radiothérapie, Institut Curie, 75005 Paris, France

**Keywords:** HER-2/neu, circulating tumour cells, prostate cancer, metastatic process

## Abstract

HER-2/neu may play a role in prostate carcinogenesis. The aim of this study was to use the expression of HER-2/neu as a molecular marker for the detection of circulating tumour cells (CTCs) in the blood of patients with prostate cancer (PC). Blood samples were collected from 42 patients with PC and nine healthy volunteers. Immunomagnetic beads were used to harvest epithelial cells from peripheral blood mononuclear cells. Total RNA was extracted and reverse transcribed before analysis by real-time PCR with HER-2/neu-specific primers. CTCs were HER-2/neu positive in six out of 11 (54%) patients with metastatic disease and in three out of 31 (9.6%) patients with localised PC (*P*=0.004). In blood samples from nine healthy volunteers, we detected no expression of HER-2/neu. The present method appears to be minimally invasive, highly sensitive and a specific approach for detecting CTCs in PC. Furthermore, it may help better target HER-2/neu in advanced PC.

Prostate cancer (PC), the most common cancer in men in western countries, presents a dynamic process in which different clinical and biological phases in both the natural and treated history of the disease may be characterised (Scher and Heller, 2000). The pathways that underlie tumour progression, invasion and resistance to treatment in each clinical state are being increasingly understood, and novel biologic agents that target these pathways are in development or are now available for clinical testing. On the other hand, establishment of new biomarkers is needed to identify high-risk patients as candidates for new (adjuvant) therapies and to find new surrogate endpoints to assess the efficacy of these treatments (De Marzo *et al*, 2001).

Among serum markers of prostate carcinogenesis, the detection of circulating tumour cells (CTCs) by RT-PCR for prostate-specific antigen (PSA) and prostate-specific membrane antigen (PSMA) mRNA proved to be positive in both peripheral blood and bone marrow (BM). The level of CTCs detected in this setting ranges from 0 to 72% in patients with clinically organ confined (T1-2) disease and from 25 to 100% in patients with distant metastases (Israeli *et al*, 1994; Katz *et al*, 1994; Ghossein *et al*, 1995; Seiden *et al*, 1995; Sokoloff *et al*, 1996; Corey *et al*, 1997; Wood and Banerjee, 1997; Mejean *et al*, 2000; Hara *et al*, 2002). However, the major question of the specificity of the RT-PCR approach remains unanswered because of illegitimate transcripts in lymphocytes or other blood cells.

The detection of CTCs using more specific markers such as overexpression of HER-2/neu may be more attractive.

The HER-2/neu oncogene is located on chromosome 17q21 and it encodes a 185 kDa transmembrane tyrosine kinase receptor of the epidermal growth factor receptor family (Schechter *et al*, 1985; Yamamoto *et al*, 1986). Amplification of the HER-2/neu gene and/or overexpression of the protein are found in a variety of human cancers and a number of clinical studies have attempted to correlate its presence with poor patient prognosis (Hynes, 1993; Hynes and Stern, 1994). In breast cancer, HER-2/neu amplification or overexpression, according to standardised criteria, has been described as a marker predicting both response to treatment and poor prognosis with nodal metastases (Slamon *et al*, 1987; Menard *et al*, 2000; Yamauchi *et al*, 2001). Targeting HER-2 using a humanised monoclonal antibody (trastuzumab, Herceptine®) in combination with chemotherapy confers a survival benefit for patients with metastatic breast carcinoma that overexpress HER-2 (Slamon *et al*, 2001), raising the possibility of therapeutic benefits for other types of malignancies that similarly express HER-2. Although the assessment of HER-2/neu overexpression in PC varies widely due to procedural differences, evidence suggests that HER-2/neu may be important in cancer progression (Signoretti *et al*, 2000). Antitumour activity of anti-HER-2/neu antibody was shown in both androgen-dependent and -independent human xenograft models (Agus *et al*, 1999). Preliminary clinical trials of trastuzumab have been performed in metastatic PC (Small *et al*, 2001; Morris *et al*, 2002). The results of these trials underscored the requirement to better access HER-2/neu expression in order to identify subgroups of patients who may benefit from HER-2/neu targeting.

The aim of the present study was to use the expression of HER-2/neu as a molecular marker for the detection of cancer cells in the blood of 42 patients with PC and various clinical stages. Indeed, compared to other molecular markers (e.g. PSA), HER-2/neu has several advantages: it plays a role in prostate tumour progression, and it represents a potential therapeutic target since a commercially available antibody exists and has been successfully tested in the setting of breast cancer. In addition, overexpression of HER-2/neu is highly specific for cancer cells and has never being reported in the blood cells. Therefore, the use of HER-2/neu avoids the problem related to illegitimate transcripts in lymphocytes or other blood cells.

## MATERIALS AND METHODS

### Patients

A total of 42 men, aged 54–81 years, with histologically confirmed PC were included in this study. Blood samples were collected before any treatment in patients with localised PC and during hormone therapy in patients with metastases. All the blood samples were taken at an interval from any biopsy or digital examination. For patients who underwent surgery, blood was collected the day before the surgical procedure. According to clinical status and treatment, patients were divided into two groups: 11 patients with distant metastasis receiving hormone therapy (group 1) and 31 patients with localised PC (group 2). Among patients with localised PC (*n*=31), 18 were treated by radical prostatectomy (patient no. 12–29) and 13 by radiation (patient nos. 30–42) (see Tables 1

**Table 1:** Clinical, pathological and biological status of 11 patients with metastatic prostate carcinoma (group 1)

						**Circulating tumour cells**
**Patient no.**	**Age**	**Score of gleason**	**M**	**Plasmatic marker: PSA^b^,^c^**	**Treatment**	**Status HER-2/neu: RT-PCR (LightCycler)**
1	79	2	+	15	HT	−
2	67	7	+	0.8	HT	+
3	67	7	+	88	HT	+
4	79	7	+	0.9	HT	−
5	74	7	+	2.38	None	+
6	74	4	+	0.1	HT	−
7	81	4	+	2.9	HT	+
8	72	7	+	3.1	HT	−
9	68	7	+	82.3	HT	−
10	78	5	+	508	HT	+
11	80	4	+	492	HT	+

a HT=hormonotherapy; CTC=circulating tumour cell; PSA=prostate-specific antigen.

b Normal value for PSA: <5 ng ml^−1.^

c PSA kit (Kryptor-PSA, Brahms, Germany).

and 2

**Table 2:** Clinical, pathological and biological status of 31 patients with localised prostate carcinoma before radical prostatectomy (patients 12–29) and before treatment by radiation therapy (patients 30–42)

							**Circulating tumour cells**
**Patient no.**	**Age**	**Score of gleason**	**T**	**N**	**M**	**Plasmatic marker: PSA^b^**	**Status HER-2/neu: RT-PCR (LightCycler)**
12	62	6	2b	X	0	16.3	−
13	74	7	3b	1	0	17.9	−
14	68	5	2a	X	0	8	−
15	66	5	3a	X	0	8.8	−
16	79	6	3a	X	0	12.2	−
17	64	7	2a	X	0	9.1	−
18	67	5	2b	X	0	5.9	−
19	69	6	2b	X	0	13.3	−
20	62	7	3b	X	0	12.5	−
21	60	6	2b	X	0	7.1	−
22	76	6	2b	X	0	11	−
23	72	6	2a	X	0	7	−
24	62	6	2b	X	0	0.9	−
25	66	9	3b	X	0	8.5	−
26	57	7	3b	X	0	8.2	−
27	78	9	3b	X	0	6.3	−
28	65	5	2b	X	0	2.6	−
29	55	6	2b	0	0	33	−
30	78	2	1c	0	0	6.4	−
31	67	7	1c	0	0	6	−
32	74	5	1c	0	0	10	−
33	74	5	2a	0	0	7	−
34	63	6	2a	0	0	35	+
35	58	3	2a	0	0	10	−
36	75	7	2a	0	0	9	−
37	74	7	1c	0	0	13	+
38	54	6	1c	0	0	10.6	−
39	58	6	1c	0	0	5.9	+
40	57	8	2a	0	0	20	−
41	61	8	2a	0	0	8.9	−
42	66	5	1c	0	0	5	−

a HT=hormonotherapy; ND=not determined; PSA=prostate-specific antigen.

b Normal value for PSA: <5 ng ml^−1^. T=pT for patients 12–29, T=clinical and radiological results for patients 30–42; N=pN for patients 12–29, N=clinical and radiological results for patients 30–42; M=radiological results.

). For surgically treated patients, localised PC was defined histologically (pT). For patients treated by radiation therapy, PC was clinically and radiologically defined. All these patients underwent endorectal coil MRI, in order to rule out extracapsular or seminal vesicules invasion. Nine healthy male volunteers, aged 34–60 years, were selected as a negative control group.

### Blood samples and immunomagnetic separation of circulating epithelial cells

Blood samples (20 ml) were collected in heparinised tubes and stored at 4°C for a maximum of 2 h before the experiments. Epithelial cells were separated as described by Soria *et al* (1999). Briefly, PBMCs were isolated by using Ficoll/*et al*, 1999. Hypaque and resuspended in 1 ml of PBS-2% FCS. Then, 12.5 × 10^6^ prewashed immunomagnetic beads covalently coated with the BerEP4 monoclonal antibody (Dynal A.S., Oslo, Norway) were added. BerEP4 monoclonal antibody recognises an epitope on the protein moiety of 2 glycopeptides (34 and 39 kDa) expressed at the surface of epithelial cells in normal and malignant tissues (Latza *et al*, 1990). Following incubation at 4°C for 30 min, cells bound to the beads were harvested using a magnetic field. The harvested epithelial cells (HECs) were then washed six times with PBS–2% FCS. Washing efficiency was controlled by microscopic examination to verify that the samples only contained immunomagnetic bead-coated cells. Cells and beads were then stored at −80°C in two Eppendorf tubes for PCRs.

### RNA extraction

RNA was extracted from cells using the commercially available kit (RNeasy 74104, Qiagen, Hilden, Germany). Samples were stored at −80°C for up to 4 weeks before the assay.

### cDNA synthesis

The detection of HER-2/neu mRNA was performed in a two-step procedure as described by the LightCycler HER-2/neu RNA Quantification Kit (Roche). Briefly, in the first step, cDNA was prepared from RNA by reverse transcription in a final volume of 20 *μ*l in a thermal cycler (PTC-200, MJ Research). The samples were incubated at 25°C for 10 min, and then 42°C for 60 min and 94°C for 5 min. cDNA were stored at −20°C.

### PCR conditions

In the second step, a 395 bp fragment of HER-2/neu mRNA was amplified from cDNA by PCR using specific primers (LightCycler-HER2/neu RNA Quantification Kit, Roche, Mannheim, Germany). The amplicon was detected by fluorescence using a specific pair of Hybridisation Probes consisting of two different oligonucleotides labelled at the 5′-end with LightCycler-Red 640 and at the 3′-end with LightCycler-Fluorescein. Only after hybridisation to the template DNA do the two probes come in close proximity, resulting in fluorescence resonance energy transfer (FRET) between the two fluorophores measured by the LightCycler instrument. The kit included a calibrator containing a stabilised fraction of total RNA purified from an immortalised cell line constitutively expressing HER-2/neu. The efficiency of PCR was assessed with serial dilutions of this calibrator in each run. For quantification, crossing point (Cp) was used, the cycle at which PCR amplification begins its exponential phase and is considered the point most reliably proportional to initial concentration. The amount of mRNA encoding for HER-2/neu is expressed as a relative ratio to a reference gene (G6PDH) in a sample relative to the Her-2/neu : G6PDH ratio in a calibrator.

Of the total reverse transcription volume of 20 *μ*l, 4 *μ*l was used for each PCR. The polymerase amplification was performed in a total volume of 10 *μ*l. Each experiment was performed in duplicate. The thermal cycling conditions were: denaturation for 20 min at 95°C followed by amplification 10 s at 95°C, then 15 s at 65°C and 15 s at 72°C for 50 cycles, terminated by a cooling step 30 s at 40°C.

## RESULTS

### Determination of assay sensitivity

**Figure 1:**
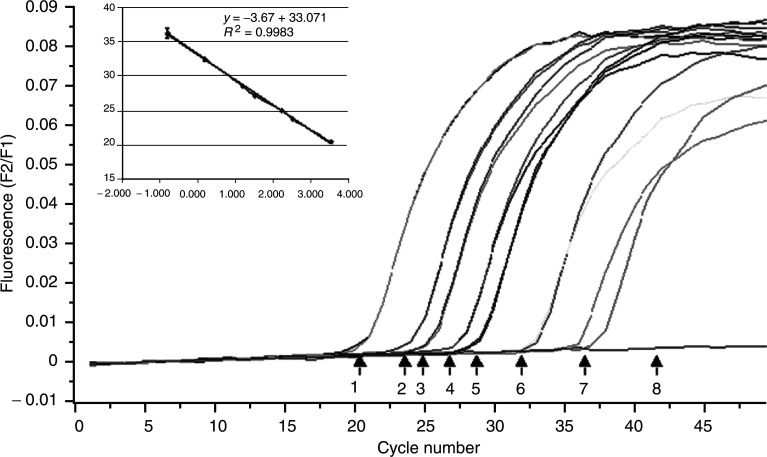
Expression of HER-2/neu RNA by real-time PCR (LightCycler). Amplification curve shows serial dilutions of an initial amount of SK-BR-3 RNA: 1 (3333 cells), 2 (333 cells), 3 (166 cells), 4 (33 cells), 5 (16 cells), 6 (1.6 cells) and 7 (0.6 cells). Inset, standard curve plot of the log of SK-BR-3 RNA cells numbers *vs* Cp. The standard curve shows seven orders of linear dynamic range (*y*=−3.67*x*+33.071; *R*^2^=0.9983).

We analysed the sensitivity of the present method by serial dilutions of SK-BR-3 cell line, which is used as a reference for overexpression of HER-2/neu (Pergram *et al*, 1997). The lower limit of detection was that of ⩽1 cell ml^−1^ of blood (Figure 1

).

### Detection of HER-2/neu transcripts in CTCs in the blood of healthy volunteers

In blood samples from nine healthy volunteers, we detected no expression of HER-2/neu.

### Detection of HER-2/neu transcripts in CTCs in the blood of patients with PC

#### Group 1 (metastatic PC)

Group 1 included 11 patients with metastatic PC. The median age of these patients was 74 years (range, 67–81). Of these 11 patients, six (54%) were positive for the detection of HER-2/neu.

#### Group 2 (localised PC)

Group 2 included 31 patients with T1c-T3b PC from whom blood was collected before treatment by radical prostatectomy or radiation therapy. The median age was 66.5 years (range, 54–79). Of these 31 patients, three (9.6%) were positive for the detection of HER-2/neu.

Tables 1 and 2 summarise the biological, clinical and pathological status of patients at sampling time.

**Figure 2:**
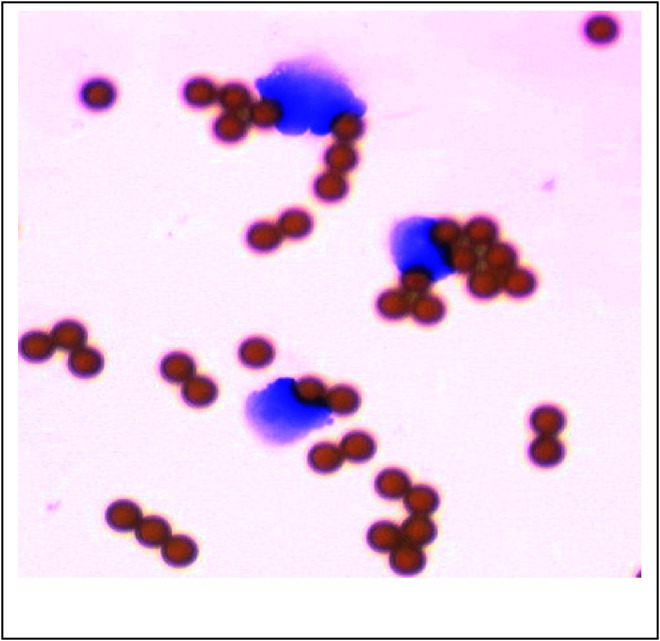
Carcinoma cells isolated with BerEP4-coated immunomagnetic beads from patient no. 34, coloration by May–Grünwald–Giemsa (CTCs isolated from a patient with localised prostate carcinoma before treatment by brachytherapy, this was an HER-2/neu-positive patient).

The specific association of HER-2/neu transcripts with the presence of epithelial cells in patients with PC was demonstrated by the absence of contaminating lymphocytes in HEC samples. The washing efficiency and lack of lymphocytic contamination were controlled for each sample by microscopic examination to verify that samples only contained immunomagnetic bead-coated cells (Figure 2

shows an example of this cytologic analysis from patient #34).

We studied the relationship of HER-2/neu positivity in CTCs of patients with different clinical status. The rate of HER-2/neu detection in CTCs of patients with metastatic PC was significantly higher than that of patients with localised disease (Fisher's exact test, *P*=0.004) (see Table 3

**Table 3:** HER-2/neu expression in CTCs of prostate cancer patients according to clinical status

**Patient characteristics**	**Number of patients**	**Positive cases %**
Metastatic PC (group 1)	11	6(54%)
Localised PC (group 2)	31	3(9.6%)*

* *P*=0.004 by Fisher exact test. CTC=circulating tumour cell; PC=prostate cancer.

).

## DISCUSSION

RT-PCR of several prostate-related genes (PSA, PSMA) as well as immunohistochemical assays (PSA, CK 18) have been used to detect circulating PC cells, but none of these techniques is currently applied in the clinical setting. To our knowledge, HER-2/neu has not been used for detecting CTCs from PC. In the present study, we have combined immunomagnetic separation of circulating epithelial cells with RT-PCR detection of the HER-2/neu transcripts, in order to increase the specificity and better characterise the molecular profile of CTCs. Nevertheless, the complexity of the present technique should be kept in mind, if its implementation in clinical practice is envisioned. Furthermore, it is important to highlight that the present procedure cannot ensure that the circulating cancer cells detected are PC cells. Indeed, such CTC could be from another epithelial cancer expressing Her2/neu (e.g. bladder cancer). Nevertheless, this is not a frequent clinical situation, especially in patients with radiological and clinical follow-ups.

Evidence suggests that co-operative signalling via c-erbB receptors (such as, HER-1, HER-2/neu, HER-3 and HER-4) can regulate many key processes such as angiogenesis and invasion in most cancers (Eccles, 2001; O-charoenrat *et al*, 2002). *In vitro*, HER-2/neu induced metastatic capacities, when it was transfected into a PC cell line (Zhau *et al*, 1999). When evaluated by immunohistochemistry (IHC), HER-2/neu expression varies largely from one study to another because of methodologic differences and heterogeneity of PC (Gu *et al*, 1996; Lara *et al*, 2002). The published incidence of HER-2/neu overexpression is typically in the range of 25–40% and 60–80%, respectively, in localised PC (Sadasivan *et al*, 1993; Ross *et al*, 1997; Fossa *et al*, 2002) and in metastatic PC (Osman *et al*, 2001; Morris *et al*, 2002). An elevation of serum HER-2/neu was also observed for detection of the extracellular domain of the protein by immunoradiometric assay in patients with advanced cancer stages (Arai *et al*, 1997). However, alterations in the HER-2/neu expression as a tumour progression marker from localised to metastatic PC have yet to be fully established. Nevertheless, *in vitro* and clinical studies have linked HER-2/neu expression to the development of late androgen-independent disease (Craft *et al*, 1999; Signoretti *et al*, 2000). These data suggest that HER-2 overexpression would be higher if a tumour sample can be obtained from patients with androgen-independent disease. This is a difficult challenge, because very few of these patients have diseases that can be biopsied readily, because metastatic PC involve mostly the skeleton.

To date, cancer-targeting therapy by trastuzumab is only indicated for metastatic breast cancer patients with an overexpression of HER-2/neu by IHC (3+), while in PC this has not yet been evaluated.

In the present study, we evaluated the frequency of the expression of HER-2/neu in CTCs of patients with localised PC *vs* patients with metastatic PC. HER-2/neu was detected in 54% of CTCs of patients with a metastatic disease and in 9.6% of CTCs of patients with localised PC (*P*=0.004). This result suggests a potential value for HER-2/neu as a marker of tumour progression even when evaluated in CTCs. Indeed, the precise phenotypic profile of the CTCs truly responsible for clinical metastasis is not known to date. Table 4

**Table 4:** RT-PCR or PCR detection of CTC and BM micrometastases in PC

			**RT-PCR or PCR positive/total(%)**	
**Author**	**Marker**	**Sample**	**Localised PC**	**Metastatic PC**
Katz *et al*	PSA mRNA	Blood	25/65 (38%)	14/18 (78%)
Israeli *et al*	PSA mRNA	Blood	0/18 (0%)	6/24 (25%)
	PSMA mRNA	Blood	13/18 (72%)	16/24 (67%)
Seiden *et al*	PSA mRNA	Blood	3/41 (7%)	11/35 (31%)
Ghossein *et al*	PSA mRNA	Blood	4/25 (16%)	26/76 (34%)
Sokoloff *et al*	PSA mRNA	Blood	43/69 (62%)	29/33 (88%)
	PSMA mRNA	Blood	12/69 (17%)	13/33 (39%)
Corey *et al*	PSA mRNA	Blood	12/63 (19%)	6/13 (46%)
		BM	45/63 (71%)	10/13 (77%)
Wood and Banerjee	PSA mRNA	BM	39/86 (45%)	NA
Mejean *et al*	PSA mRNA	Blood	NA	9/9 (100%)
Laribi *et al*	Prostasin mRNA	Blood	18/69 (26%)	17/27 (63%)
Hara *et al*	PSA mRNA	Blood	1/41 (2.4%)	7/14 (50%)
	PSMA mRNA	Blood	2/41 (4.9%)	9/14 (64%)
	PSCA mRNA	Blood	0/41 (0%)	7/14 (50%)
Present series	HER-2/neu mRNA	Blood	3/31 (9.6%)	6/11 (54%)

CTC=circulating tumour cell; BM=bone marrow; PC=prostate cancer; PSA=Prostate-specific antigen; PSMA=prostate-specific membrane antigen; PSCA=prostate stem cell antigen.

summarises our results as compared to previously published manuscripts.

Among the 31 patients with localised PC, 16 patients were classified as ‘low-risk patients’ (serum PSA ⩽10 and Gleason score ⩽6) and 15 patients were classified as ‘intermediate or high-risk patients’ (PSA: 10–20 or Gleason score=7 and PSA >20 or Gleason score: 8–10). One of 16 patients and two of 15 patients are positive for the detection of HER-2/neu in these groups, retrospectively. To establish whether patients with localised disease and HER-2/neu-positive CTCs are at a higher risk of recurrence will require additional follow up. The status of HER-2/neu in the primary tumours of the patient population is unfortunately not available and will be very difficult to obtain since many patients were referred from outside centres.

In summary, the presence of HER-2/neu in CTCs of patients with PC is more frequent in patients with metastases than those with localised disease. This technique may be of great assistance to identify patients who may benefit from targeting HER-2/neu in the clinic. In the future, additional studies should evaluate a panel of different biomarkers in CTCs. This panel may include biomarkers (such as MMP2, MMP9 or E-cadherine) able to better predict the biological behaviour of CTCs (i.e. clinical metastasis). One limitation to this approach will be the amount of CTCs and therefore the corresponding RNA concentration available for such studies.
